# A review on polysaccharide-based jelly: Gell food

**DOI:** 10.1016/j.fochx.2024.101562

**Published:** 2024-06-18

**Authors:** Aoxue Hu, Yu Liu, Shengjun Wu

**Affiliations:** a Jiangsu Key Laboratory of Marine Bioresources and Environment/Jiangsu Key Laboratory of Marine Biotechnology, Jiangsu Ocean University, Haizhou 222005, China; b Co-Innovation Center of Jiangsu Marine Bio-industry Technology, Haizhou 222005, China

**Keywords:** Gel food, Jelly, Polysaccharide

## Abstract

The prevalence of gel foods in the food industry has grown significantly due to their high water content, low calorie content, and ability to enhance satiety. This review focuses on jelly powder, the earliest form of gel food in the current food industry. Jelly is the earliest form of the gel-food, dating back to the Northern Song dynasty in China, and it relies on gelatinizing and aging of starch to form a gel. With the development of technology, jelly gradually evolved to rely on gel form of food additives. Jelly is divided into starch jelly and non-starch jelly according to their different gel formation. The development status of the two kinds of jelly is also summarized. Additionally, the current research status of these materials is summarized to broaden the understanding of gel food and offer valuable insights for future research in this field.

## Highlights

• The main raw materials used in gel foods are starch such as pea starch, corn starch and sweet potatoe starch.
• The main raw materials used in gel foods are non-starch polysaccharide such as konjac gum, carrageenan, and xanthan gum.
• Research status of starch jelly and non-starch jelly in gel food is summarized.

## 1 Introduction

In the food industry, many food products are in gel form. Recently, gel-based foods have gained popularity due to their high water content, low calorie content, and satiety-enhancing properties (Xueer et al., 2023). Colloids generally refer to carbohydrate polymers, with their chemical structures primarily composed of numerous monomers with more than two reactive positions. Most colloids utilize different monosaccharides or amino acids as structural units, which then combine through glycosidic or peptide bonds to form polysaccharides, peptides, or their derivatives (Xi, Anqi, Dan, Yurong, & Lijun, 2021). A gel is a unique dispersion system where colloidal particles in a solution connect with each other under specific conditions, forming a spatial network structure. These structures are filled with liquid as a dispersion medium (Wang, Yang, Gao, Zhang, & Hu, 2016). Gel formation can effectively alter the shape and texture of food, improving its water-holding capacity, thickening ability, and particle adhesion, while also providing a viscous and palatable taste (Jia, 2021). The gel, located between solid and liquid states, is also known as gelatin, and its elasticity, hardness, and other characteristics are crucial factors influencing the processing and molding of gel-based food.

Jelly is the earliest form of the gel-food, dating back to the Northern Song dynasty in China, and it relies on gelatinizing and aging of starch to form a gel. With the development of technology, jelly gradually evolved to rely on gel form of food additives. Jelly is divided into starch jelly and non-starch jelly according to their different gel formation (Table 1). The main raw materials used in gel foods are starch such as pea starch, corn starch and sweet potatoe starch and non-starch polysaccharide such as konjac gum, carrageenan, and xanthan gum. Additionally, the current research status of these materials is summarized to broaden the understanding of gel food and offer valuable insights for future research in this field.

**Table 1:** Gel raw materials of starch gel and non-starch gel.

Class	Gel raw materials
Starch jelly	Pea starch
	Corn starch
	Sweet potato starch
Non-starchy jelly	Konjac gum
	Xanthan gum
	Carrageenan

## 2 Starch jelly

### 2.1 Definition of starch jelly

**Fig. 1:**
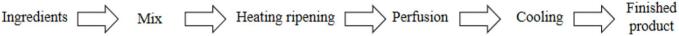
Starch gel preparation process.

Starch is a key source of carbohydrates for humans and a primary energy source. Starchy foods are integral to our daily diet, providing the necessary carbohydrates for our bodies. When starch is gelatinized and aged in water, it forms a nearly transparent gel with a certain level of elasticity and gel strength (Zhang et al., 2024). Starch gel food is a traditional Chinese cuisine with distinctive flavor characteristics. It is made from grains, potatoes, or bean starch. The starch is cooked, gelatinized, molded with added seasonings, color, and flavor, resulting in an appetizing and visually appealing product that is highly cherished by the Chinese population (Fig. 1). It enjoys a substantial consumer market within the country.

### 2.2 Formation of starch jelly

Starch gel food refers to a category of foods that form a gel after starch gelatinization. These foods have high starch content, moisture content, and water activity. When starch undergoes gelatinization, it transforms into a translucent gel with a specific level of elasticity and strength (Dai et al., 2024). Starch is a hydrophilic colloid, and the formation of starch gel primarily involves the ordered winding of amylose molecules. After gelatinization, starch granules absorb water and expand, causing the dispersion of amylose from the starch grains. During the subsequent cooling process, the amylose molecules continually intertwine with one another in a double helix formation, ultimately producing a continuous three-dimensional network structure. Amylopectin, on the other hand, forms a dispersed phase. As a result, the two phases become incompatible and create a heterogeneous mixed system (Li, Xue, Li, & Sun, 2022).The essence of starch gel lies in the transition of starch microstructure from an ordered state to a disordered state. This transition can be divided into three stages (Deda et al., 2023): In the first stage, prior to reaching the gelatinization temperature, water enters the starch grains through the pores and adsorbs or combines with the hydrophilic groups in the amorphous parts. This process causes a reversible expansion.

In the second stage, as the water temperature reaches the gelatinization temperature, a significant amount of water penetrates the starch grains, resulting in rapid swelling. Water molecules enter the microcrystalline structure of the starch, disrupting the arrangement and increasing viscosity. This irreversible expansion process leads to the formation of starch paste.

The third stage involves the cooling process, during which the starch chains wind into an amorphous state.

The gelatinization of starch not only enhances digestibility, but also effectively controls the quality and texture of starch-based foods (Li et al., 2024). Commonly used raw materials for starchy jelly preparation include peas, corn, and sweet potatoes.

#### 2.2.1 Pea starch

Pea, an annual climbing herb from the legume family, is native to western Asia and the Mediterranean coastal region. It is not only rich in various nutrients required by the human body, such as protein (17.35%–25.30%), starch (39.08%–55.42%), vitamins, minerals, etc., but also contains gibberellin, anti-bactericidal acid, and other substances with anti-inflammatory and bactericidal effects. These compounds promote human metabolism, giving peas high edible value (Okon et al., 2023). Starch, which is a by-product of pea protein extraction, is a significant component of peas. Smooth pea starch particles typically have a diameter in the range of 2–40 μm, with most being ovate in shape, although a few are spherical or irregular (Parastou, Nooshan, Bipin, Scott, & Yonghui, 2024). Gelatinization of pea starch occurs when it is heated to temperatures between 50 °C to 60 °C in excess water. During gelatinization, water molecules first penetrate the amorphous regions of starch, causing the granules to swell. As the temperature increases, the starch granules continue to swell, and the amylose component begins to dissolve until it is completely dissolved, resulting in the collapse of the granules that contain amylopectin. Ultimately, the starch granules disintegrate to form a starch paste. Starch has excellent palatability and rehydration properties after undergoing high gelatinization, and it is easily hydrolyzed by amylase, making it a common ingredient in convenience food processing (Dhull et al., 2024). Starch gelatinization during food processing often leads to the aging of starch. When the temperature decreases, gelatinized starch begins to regenerate, and the dissociated double helix structure reestablishes hydrogen bonds, resulting in the formation of a relatively ordered gel structure (Yang et al., 2020).

#### 2.2.2 Corn starch

Corn, a type of annual herbaceous plant belonging to the grass family, is also known as maize, bud valleys, and bud rice. It is extensively grown in China and serves as the country's primary cash crop. Compared to wheat and rice, maize exhibits strong tolerances to drought, barrenness, and cold temperatures. Corn is rich in nutrients such as protein, fat, starch, vitamins A, B1, B2, and E, as well as various minerals (Xu et al., 2024). Numerous studies have demonstrated that corn possesses various beneficial biological functions, including the reduction of blood lipids, blood pressure regulation, and improvement of intestinal function. As a result, corn is a highly nutritious food source (Zhang, Pan, Wang, Zhang, & Chen, 2022).

Corn starch, the principal constituent of corn grains, typically contains small amounts of fat and protein in commercially available forms. Depending on its amylopectin content, corn starch can be categorized into high-amylose, waxy corn starch, and ordinary corn starch. The amylose content of high-amylose corn starch accounts for more than 50%, while waxy corn starch contains approximately 100% amylopectin. Common corn starch has an amylose content of around 26% (Lv et al., 2024). Different types of corn starch possess distinct physicochemical properties and find varying applications. High-amylose corn starch films demonstrate high tensile strength and toughness, thereby effectively isolating air and significantly prolonging food storage life (Liu, Xing, Ke, & Zhou, 2024). On the other hand, corn starch paste functions as a thickener in food and exhibits low regeneration, high stability, and high transparency characteristics (Wang, Gao, & Wang, 2023).

#### 2.2.3 Sweet potato starch

The sweet potato, a member of the Spinophoraceae family, is indigenous to South America and the Greater and Lesser Antilles. It has been extensively cultivated in tropical and subtropical regions worldwide and has also had successful cultivation in northerly regions such as Heilongjiang Province in China. Studies have shown that sweet potatoes are a rich source of essential nutrients including protein, fiber, sugar, fat, B-carotene, vitamins B1, B2, and various trace elements such as sodium, magnesium, potassium, gallium, manganese, iron, and nitrogen. They also provide essential amino acids that are lacking in rice and wheat flour (Jati Ignasius Radix, Darmoatmodjo Laurensia, Suseno Thomas, Ristiarini, & Wibowo, 2022). Sweet potatoes also contain a range of bioactive compounds such as polysaccharides, proteins, polyphenols, and flavonoids. For example, American researchers have extracted dehydroepiandrosterone (DHEA), an active antioxidant, from sweet potatoes (Xu et al., 2017). DHEA has demonstrated anti-inflammatory properties and promotes intestinal cleansing, thereby reducing the risk of certain conditions such as hypertension, hyperlipidemia, cancer, and aging (Schwartz, 2022). The high content of P-carotene, vitamin C, and folic acid in sweet potatoes helps to eliminate free radicals, thus preventing arteriosclerosis and cancer. Japanese scientists have also isolated a unique protein from sweet potatoes. This protein, a combination of polysaccharides and proteins, enhances the immune system and prevents fatigue. It can also interact with inorganic salts to promote bone formation and prevent osteoporosis (Liu, Zhao, Li, & Jin, 2021). Additionally, sweet potatoes have been linked to diabetes prevention and cholesterol reduction (Jawi, Indrayani, & Sutirta-Yasa, 2015). Therefore, sweet potatoes are recognized as a highly valuable food source in the present era.The starch content of sweet potatoes is notably high, ranging from 16% to 25%, with a dry matter rate of more than 35%. Sweet potato starch is derived from fresh sweet potatoes through a series of processes including washing, crushing, screening, sedimentation, dehydration, and drying. The resulting starch is white in color. In sweet potatoes, the starch content is approximately 30%, with amylose accounting for 15% to 25% and amylopectin making up 75% to 85%. The particle shape of sweet potato starch resembles that of corn and wheat starch, characterized by larger granules that have a smooth surface without any holes. The transparency of sweet potato starch paste is comparable to that of potato starch. It should be noted that the initial temperature for starch gelatinization in sweet potatoes is lower than that of wheat and corn starch, leading to a higher viscosity in the starch paste compared to cereal starch. Currently, most sweet potato starch paste in China is utilized for the production of gel foods, while a smaller portion is used for preparation of Western medicine tablets, film-forming agents, and preservatives (Zhang et al., 2022).

### 2.3 Research status of starch jelly

Starch jelly is a gelatinous food that originated in the Northern Song Dynasty of China. It is made primarily from jelly grass, rice, sweet potatoes, and peas. The jelly's color, texture, and taste vary depending on the raw materials used. For example, jelly made from pea starch is white and flawless, while sweet potato starch produces a gray color, and potato starch creates a clear jelly. Over time, jelly has become a popular convenience food enjoyed during leisure activities and travel (Tian & Huang, 2019).

With advancements in food technology, the production of jelly has expanded beyond traditional ingredients such as rice, sweet potatoes, mung beans, and jelly grass. For instance, Yu et al. (2024) reported on the high starch content in buckwheat, with buckwheat serving as the primary raw material and quinoa replacing traditional edible alkali. By heating the mixture at 75 °C, with a raw material ratio of 5:1 g/g (buckwheat:quinoa) and a solid-liquid ratio of 3:1 mL/g, adding a composite gum of xanthan gum, carrageenan, and guar gum at a mass ratio of 4:5:4, and heating for 1 h, jelly with a uniform gel structure was successfully prepared. Xia et al. (2023) prepared tartary buckwheat jelly by heating at a solid-liquid ratio of 1:5, adding edible salt at 0.8%, and sodium dehydroacetate at 0.08%. Wang, Lv, Yang, and Chen (2023) utilized water chestnut powder as the benchmark, adding water in a ratio of 1:6, edible alkali at 1 g, and salt at 2 g, and heating for 20 min to create water chestnut jelly with a unique flavor and elastic texture. Gao et al. (2023) extracted sweet potato leaves and utilized the extract to produce jelly with an emerald green color, uniform texture, and the distinct aroma of sweet potato leaves. Huang (2022) mixed acorn starch with water and allowed it to stand for 5 min at a solid-liquid ratio of 1:8, adding white sugar at 7%, citric acid at 0.06%, and carrageenan at 0.6%. After heating on an induction cooker over low heat for 10 min with continuous stirring, acorn jelly with a pleasing color, balanced sweetness and sourness, elasticity, and a unique taste of acorn kernels was obtained. Gou et al. (2022) investigated the impact of cool grass gum, jackfruit seed starch, and monk fruit extract on the quality of black jelly made from jackfruit seeds. The study revealed that with cool grass gum solution at 60%, jackfruit seed starch at 2.5%, and monk fruit extract at 5.00%, the sensory scores and texture parameters of black jelly made from jackfruit seeds closely resembled those made from pea, potato, and corn starch. This suggests that jackfruit seed starch can be used as a raw material for producing black jelly. Wang (2021) optimized the production process for traditional pea jelly, resulting in a delicate and smooth texture, good elasticity, appealing color, and excellent taste quality. Based on this, purple potato flour and pea starch were used as raw materials, and by heating for 10 min at a ratio of two starches (1,4), a material-liquid ratio of 1:5, and a slurry water temperature of 95 °C, purple sweet potato pea jelly with a good gel effect, superior texture and flavor, satisfactory elasticity, and a smooth appearance without roughness was successfully produced (Wang, Huang, & Li, 2021). Lee et al. (2023) developed sugar-free sweet potato jelly using sweet potatoes as the raw material and investigated the quality characteristics of various sweet potato varieties. Three sweet potato varieties were utilized: *Juwangmi* (orange), *Sinjami* (purple), and *Daeyumi* (yellow). The total free sugar and glucose contents in the hydrolysate increased during enzymatic treatment. Based on sensory evaluation, the overall preference was observed for the Daeyumi, Sinjami, and Juhangmi cultivars. This study confirmed that jelly can be produced by saccharifying sweet potatoes and highlighted the significant influence of raw sweet potato properties on jelly quality characteristics. Jung and Joo (2021) studied and developed konjac jelly, incorporating noni juice with high antioxidant activity. The researchers evaluated the quality characteristics of the konjac jelly and found that the addition of noni juice resulted in higher sensory scores.

In summary, the production of starch jelly has evolved and expanded to incorporate a wide range of ingredients and techniques. Ongoing research continues to explore new possibilities for creating diverse and innovative jelly products.3.

## 3 Non-starchy jelly

### 3.1 Definition of non-starchy jelly

**Fig. 2:**
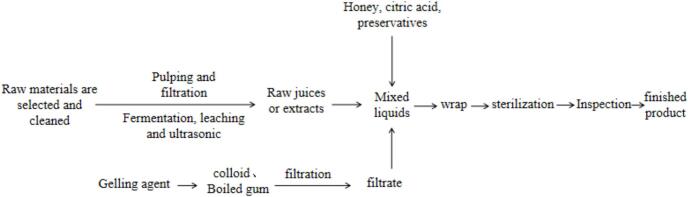
Non-Starchy preparation process.

Non-starchy jelly is a gelatinous substance that retains a significant amount of its contents after being poured out of the packaging container. As shown in Fig. 2, it is processed by sol, blending, filling, sterilization and cooling. Over time, non-starchy jelly has transformed into a light dessert option with reduced calories and fat content.

### 3.2 Formation of non-starch jelly

Non-starchy jelly is primarily composed of water, sugar, and other raw materials. Food additives such as gelling agents are often incorporated, along with optionally added fruit products, milk and dairy products, and other raw materials. In recent years, there has been a growing focus on the use of gelling agents in the production of non-starch jelly, as relying solely on water and sugar for the gelling effect is found to be inadequate. Notably, konjac gum, carrageenan, and xanthan gum have emerged as the most studied gelling agents in this regard.

#### 3.2.1 Konjac gum

Konjac gum is a non-ionic, water-soluble polymer polysaccharide extracted from the globular tubers of Konjac herbaceous plants. Its main component is konjac glucomannan (Tang, Bai, Li, & Liu, 2023). It is composed of a D-mannose and d-glucose linked by β-1,4 glycosidic bonds at 1:1.6–1:1.4 M ratio, depending on the genotypes. In addition, there are acetyl groups attributing randomly to C-6 position of the saccharide units along the molecule 1 per 19 sugar residues, and some side chains linking to mannoses by joint C-3. Side chains may exist with a degree of branching of 8%. Further, the molecular weight of KGM ranges from 500 k to Jung & Joo, 2021 k, depending on the plant source and extraction procedures (Zhan et al., 2023). Konjac glucomannan has been approved as a health food and food additive in the United States and Europe since 1994, leading to an increased demand for konjac in the international market. Japan established the “konjac research association” in 1971 to conduct research on konjac and its fine powder, achieving significant results. Japan's expertise in konjac product processing technology and equipment is internationally leading, and while it develops its own resources, it also imports raw materials from abroad, with 94% of its raw materials being imported from China.

Under certain external factors, the molecular conformation of konjac gum can change, promoting the aggregation and gelation of its molecules (García-Segovia, García-Alcaraz, Balasch-Parisi, & Martínez-Monzó, 2020). Konjac gum can form a hot, irreversible gel after undergoing a hot alkali treatment. Due to the presence of bases, the acetyl group undergoes a saponification reaction and detaches, resulting in a macromolecule with a transient active group that can be physically cross-linked with another monosaccharide residue in thermal motion to form a gel through hydrogen bonding (Yue et al., 2023). The deacetylation reaction is an induced reaction in the gelation of konjac gum.

Research has found that the thermogelation of konjac gum occurs in two stages. The first stage is driven by energy activation, causing the mildly dehydrated konjac glucomannan chain to locally unfold with a loose structure. In the second stage, the random coils transition into self-assembled network configurations, forming junction regions composed of acetyl-free moieties. This transition triggers significant agglomeration as the temperature increases, resulting in the formation of complex supramolecular structures with hysteresis and ultimately forming gels (Huang, Liu, Pei, Li, & Li, 2020).

In general, the apparent viscosity of konjac gum increases with the increase in its relative molecular weight. The viscosity also increases with concentration, and the growth rate is faster at higher concentrations. However, the viscosity of konjac gum aqueous solution of a certain concentration decreases with an increase in temperature, and this relationship is not linear. In terms of compounding properties, konjac gum exhibits synergy when mixed with xanthan gum and carrageenan (Huang, Takahashi, Kobayashi, Kawase, & Nishinari, 2002).

#### 3.2.2 Xanthan gum

Xanthan gum, also known as Hansheng gum, is a negatively charged exopolysaccharide derived from microbes. Its development originated in the United States and later expanded to France, Japan, and the United Kingdom. Currently, there is a global production of approximately 20,000 tons of food-grade xanthan gum, with an additional 20,000 tons used for industrial purposes. Over the past few decades, the demand for xanthan gum has grown steadily at a rate of 10% annually. Its versatility and relatively high cost make it a highly sought-after microbial polysaccharide. Xanthan gum is characterized by a chemical structure in which the side chains are connected to the main chain through 1,3-glycosidic bonds. The non-covalent bonds between the main and side chains predominantly consist of hydrogen bonds, resulting in a rod-like double helix structure (Zhou, Huang, & Zhang, 2008). It exhibits excellent thickening properties, forms a weak gel structure in aqueous solutions, and demonstrates good water solubility, thermal stability, and acid-base stability. Consequently, it is commonly employed as a thickener, emulsifier, and stabilizer (Asase & Glukhareva, 2024). Natural xanthan gum adopts a complete double helix structure, although upon heating, the coiled chains unfold into a disordered, curly shape. As the temperature decreases, the coiled chains and double helix structure coexist (Tan, 2010). Initial studies suggested that xanthan gum had limited gel-forming capabilities in aqueous solutions. However, subsequent research has demonstrated that gels can be formed when the solution temperature exceeds the conformational transition temperature of xanthan gum (40–50 °C) (Sriprablom, Luangpituksa, Wongkongkatep, Pongtharangkul, & Suphantharika, 2019). Moreover, xanthan gum and other hydrocolloids can form effective compound ligands that cater to various manufacturing requirements. For instance, the combination of xanthan gum with carrageenan and konjac gum enhances the gel elasticity, cohesion, and water-holding capacity of the compound system (Bhatia et al., 2024).

#### 3.2.3 Carrageenan

Carrageenan is an anionic polysaccharide derived from seaweed, with a chemical structure consisting of 1,3-β-d-galactopyranose and 1,4-α-D-galactopyranopyranose as repeating disaccharide units. These units are linked alternately to form linear galactan sulfate (Anna et al., 2022). Based on the different forms and sources of sulfate binding, carrageenan can be classified into eight types (Jiang, Zhang, Ni, & Shao, 2021). Among these types, Kappa-type and Lota-type carrageenans form a series of gel textures when their thermal solutions are cooled to 40–70 °C in the presence of cations. Carrageenan gels display hysteresis and variations between the environment and the melting temperature. While stable at room temperature, these gels melt when heated above 5–20 °C of the gel temperature. Notably, the gel strength and texture of carrageenan can be affected by hydrolysis through heating and cooling, particularly in acidic products (Covis et al., 2016).

The presence of ionic components in a food system plays a crucial role in optimizing the utilization of carrageenan. For instance, kappa-type carrageenan forms a hard and brittle gel when reacted with potassium ions. On the other hand, lota-type carrageenan utilizes sodium ions to create bridges between adjacent chains, resulting in a soft and elastic gel. The presence of these ions significantly affects the hydration temperature, environment, and melting temperature of carrageenan (Velde, Knutsen, Usov, Rollema, & Cerezo, 2002). Furthermore, the addition of salt can increase the gel point of lota-type carrageenan, converting the solution into a reversible gel with a distinct gelling point. This property is harnessed in the production of cold salad dressings.

In terms of jelly production, agar is commonly used as a gelling agent due to its elasticity. However, it is expensive and time-consuming to produce. Gelatin jelly, on the other hand, requires a low-temperature environment. Pectin jelly has a high sugar content and necessitates a specific pH environment (Luo, Chen, Meng, Huang, & He, 2003). Comparatively, carrageenan jelly exhibits good elasticity and does not separate water. In actual production, carrageenan is often combined with other hydrocolloids such as konjac gum and xanthan gum to enhance the elasticity, reduce water separation, and improve taste in jelly products (Hou et al., 2013).

### 3.3 Current research on non-starch jelly

Jelly is appreciated by consumers of all ages due to its unique texture. On the subject of consumption upgrading, there is a new trend emerging in snack consumption that emphasizes natural, nutritious, healthy, and personalized choices. Various examples of this trend include jelly made from exceptional raw materials, such as lactic acid bacteria fermented purple potato jelly (Tang, He, & Cheng, 2023); white fungus multi-candy jelly (Wang, Gao, & Wang, 2023); cherry jujube peanut cardinal iron supplement jelly (Wei et al., 2023); persimmon jelly (Yang et al., 2023); honeysuckle pear jelly (Zhang et al., 2023); orange broccoli jelly (Han, Wang, Gan, Zhang, & Chen, 2023); wakame jelly (Wu & Yu, 2023); walnut green peel pectin low-sugar jelly (Lv et al., 2023); and lotus leaf grapefruit jelly (Lv, Chen, et al., 2023), among others.In recent years, there has been significant progress in research on non-starch jelly with a focus on its potential in healthcare. Fajriyani, Anwar, and Noviasari (2024) utilized beetroot and red dragon fruit peel as raw materials, incorporating natural pigments as colorants, to develop a jelly that contains nutrients and high fiber. This jelly has the potential to help individuals regulate their blood sugarlevels. Spinei and Oroian (2023) examined the fundamental components of grape pomace and extracted its fermentation broth. They utilized this extract along with gelatin to create grape pomace jelly with a total phenolic content of 156 mgGAE/g. Szydłowska, Zielińska, Sionek, and Krajewska (2023) investigated the impact of probiotics, prebiotics, and different types of jelly agents on the quality attributes of mulberry jelly. Their findings revealed that the addition of *Lactobacillus plantarum* O21 and prebiotics, such as inulin and agar-agar, to the jelly formulation resulted in the proliferation of lactic acid bacteria. Furthermore, lactic acid fermentation positively affected the total anthocyanin concentration, leading to mulberry jelly with high levels of lactic acid bacteria, excellent organoleptic quality, and advantageous antioxidant properties. Wilawan, Kwanhian, and Yusuf (2023) developed a gummy jelly suitable for diabetics. This jelly contains Nipah palm vinegar powder and Nipah palm syrup, with an in vitro glycemic index of 27.4. Saachi, Priyanka, Dilip, Lakshmi, and Avanish (2023) explored the utilization of an underutilized fruit to produce a flavorful jelly. Their research indicated that the ideal composition for this jelly includes 40–50% pulp, 0.5–1% pectin, and 0.3–0.5% citric acid. Feng et al. (2023) developed probiotic gummies using *Bacillus coagulans*, *Bacillus claucheri*, and *Bacillus subtilis*. The addition of probiotics enhanced the total phenolic content and antioxidant capacity of the gummies. These gummies maintained their probiotic properties for at least 90 days of storage. Juliyarsi, Sandra, Melia, Setiawan, and Nurhayati, and Pangestu, S. (2023) successfully enhanced the functional properties of dadiah jelly using *Clitoria ternatea* flower extract, resulting in improved antioxidant activity and total phenolic content. Elgamily, El-Sayed, El-Sayed, and Youssef (2023) discovered that jelly made from a natural prebiotic grape seed extract, combined with Lactobacillus rhamnobacteria, exhibited an anticariogenic effect. Brindyukova, Yerenova, Syzdykova, Abdiyeva, and Tlevlessova (2023) utilized watermelon and melon as raw materials to produce jelly, incorporating red monk fruit powder. The study demonstrated that this jelly has a shelf life of up to 30 days when stored at 6 °C and is resistant to mold. Baek, Ryu, and Paik (2023) prepared citrus peel jelly with high antioxidant activity. They capitalized on the flavonoids, dietary fiber, and phenols present in citrus peel. Hung et al. (2023) addressed the environmental concerns associated with the disposal of pomelo peels by recycling and reusing grapefruit peels to create yellow and white peel jelly. Consumer sensory evaluation indicated high scores for all sensory attributes, with acceptance rates of 62% for yellow peel jelly (PYPJ) and 92% for white peel jelly (PWPJ). Further improvements are needed in PYPJ to enhance consumer acceptance. Spinei & Oroian, 2023 et al. (2021) developed a jelly suitable for individuals with Parkinson's disease, utilizing *Mucuna pruriens* seed extract as the main active ingredient. Additionally, Figueroa et al. (2019) formulated jelly containing apple fiber, bamboo fiber, and psyllium fiber, which exhibited favorable rheological, mechanical, and color properties after refrigeration for at least 30 days. The product received high ratings for its structure, stability, appearance, and flavor.

## 4 Summary and outlook

Jelly is divided into starchy and non-starchy groups, pea starch, corn starch and sweet potato starch which are commonly used to make starch jelly, and konjac gum, Carrageenan and xanthan gum which are commonly used to make non-starch jelly are introduced. Not only do the two types of jelly differ in their ingredients and processes, but they also differ in their consumer groups. Starchy jelly is more filling and is more common on the Chinese table, non-starchy jelly is like a snack food and is being developed for health.

With the advancement of living standards, there has been a corresponding shift in people's dietary habits. The traditional high-fat, high-protein, high-calorie diet has given way to a low-fat, high-fiber dietary structure that is now considered crucial in maintaining a balanced diet. Gel food, specifically non-starch polysaccharide-based products such as jellies, offer a low-calorie, low-fat alternative while still providing a delightful taste and nutritional benefits. As production technology continues to evolve and lifestyles change, there is an anticipated increase in consumer demand for gel food.

## CRediT authorship contribution statement

**Aoxue Hu:** Writing – original draft, Methodology, Investigation, Formal analysis, Data curation. **Yu Liu:** Writing – original draft, Visualization, Validation, Software, Investigation. **Shengjun Wu:** Writing – review & editing, Supervision, Resources, Project administration, Funding acquisition, Conceptualization.

## Declaration of competing interest

The authors declare that they have no known competing financial interests or personal relationships that could have appeared to influence the work reported in this paper.

## Data Availability

The authors do not have permission to share data.
